# Supplementary material for: Discovery of a Small Molecule Activator of Slack (Kcnt1) Potassium Channels That Significantly Reduces Scratching in Mouse Models of Histamine‐Independent and Chronic Itch
Source: Adv Sci (Weinh). 2024 Feb 13;11(15):2307237. doi: 10.1002/advs.202307237 (PMC11022729; doi:10.1002/advs.202307237)
Supplement: Supplementary file 1 — Supporting Information [file ADVS-11-2307237-s001.pdf]

ADVANCED SCIENCE
Open Access

Supporting Information

for *Adv. Sci.*, DOI 10.1002/advs.202307237

Discovery of a Small Molecule Activator of Slack (Kcnt1) Potassium Channels That Significantly Reduces Scratching in Mouse Models of Histamine-Independent and Chronic Itch

Annika Balzulat, W. Felix Zhu, Cathrin Flauaus, Victor Hernandez-Olmos, Jan Heering, Sunesh Sethumadhavan, Mariam Dubiel, Annika Frank, Amelie Menge, Maureen Hebchen, Katharina Metzner, Ruirui Lu, Robert Lukowski, Peter Ruth, Stefan Knapp, Susanne Müller, Dieter Steinhilber, Inga Hänelt, Holger Stark, Ewgenij Proschak and Achim Schmidtko**

Supporting Information

Discovery of A Small Molecule Activator of Slack (Kcnt1) Potassium Channels That Significantly Reduces Scratching in Mouse Models of Histamine-Independent and Chronic Itch

Annika Balzulat, W. Felix Zhu, Cathrin Flauaus, Victor Hernandez-Olmos, Jan Heering, Sunesh Sethumadhavan, Mariam Dubiel, Annika Frank, Amelie Menge, Maureen Hebchen, Katharina Metzner, Ruirui Lu, Robert Lukowski, Peter Ruth, Stefan Knapp, Susanne Müller, Dieter Steinhilber, Inga Hänelt, Holger Stark, Ewgenij Proschak, and Achim Schmidtko**

This file includes:

Figures S1 to S12

Tables S1 to S3

Supplemental protocols for synthesis of compounds

References for Supporting Information

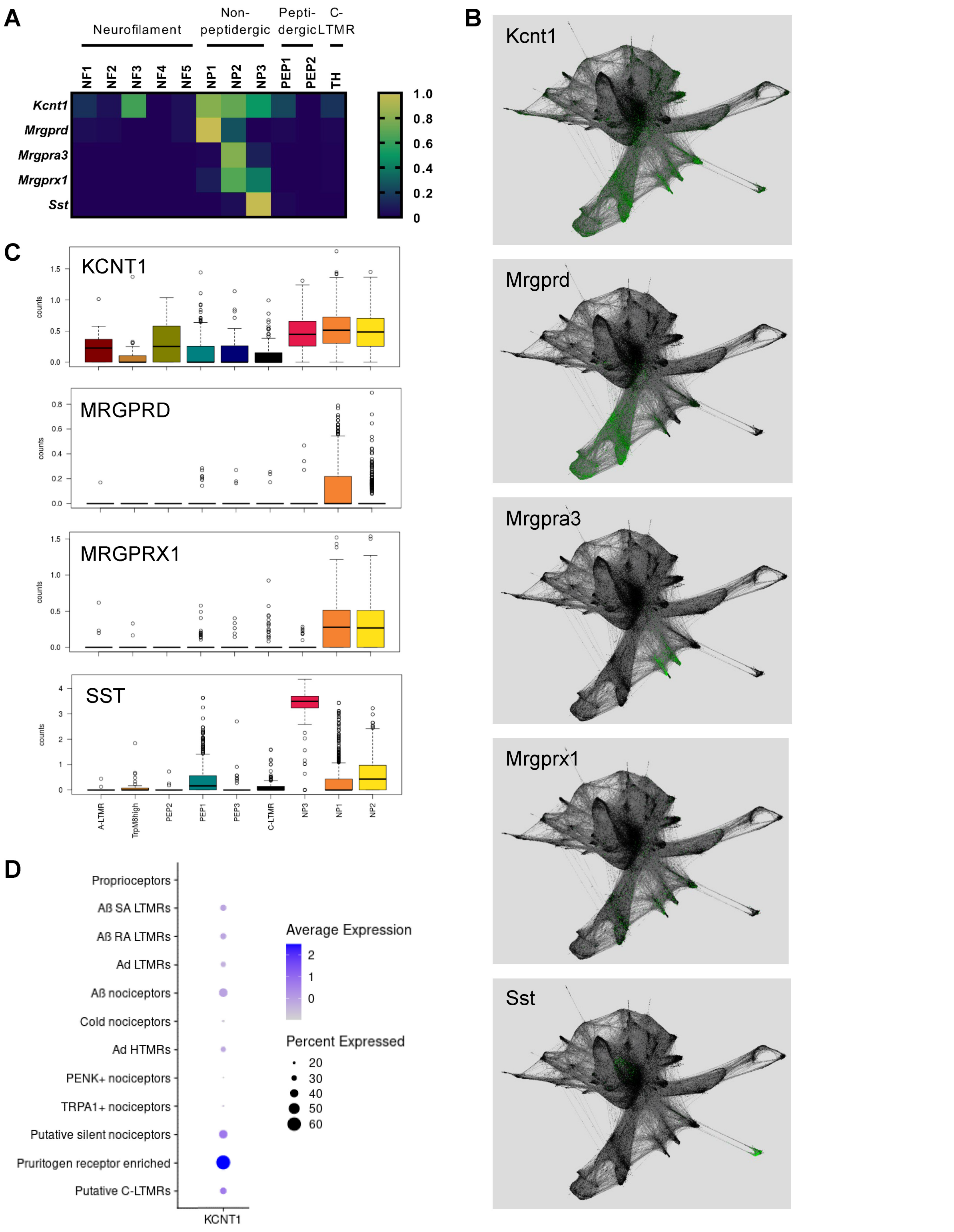

Figure S1. Expression of Kcnt1 and critical itch receptors across sensory neuron subsets. Presented are data from published single-cell RNA-seq studies. A) Expression pattern in mouse DRG neurons (6-8 week old).[1] B) Expression pattern in mouse DRG neurons (embryonic day 11.5 to postnatal day 42) presented as a force-directed layout.[2] Downloaded from:

https://kleintools.hms.harvard.edu/tools/springViewer_1_6_dev.html?datasets/Sharma2019/all. C) Expression pattern in non-human primate DRG neurons (5-14 year old).[3] Downloaded from: https://ernforsgroup.shinyapps.io/macaquedr. D) Expression pattern in human DRG neurons (24-65 year old).[4] Downloaded from: https://sensoryomics.shinyapps.io/RNA-Data/.

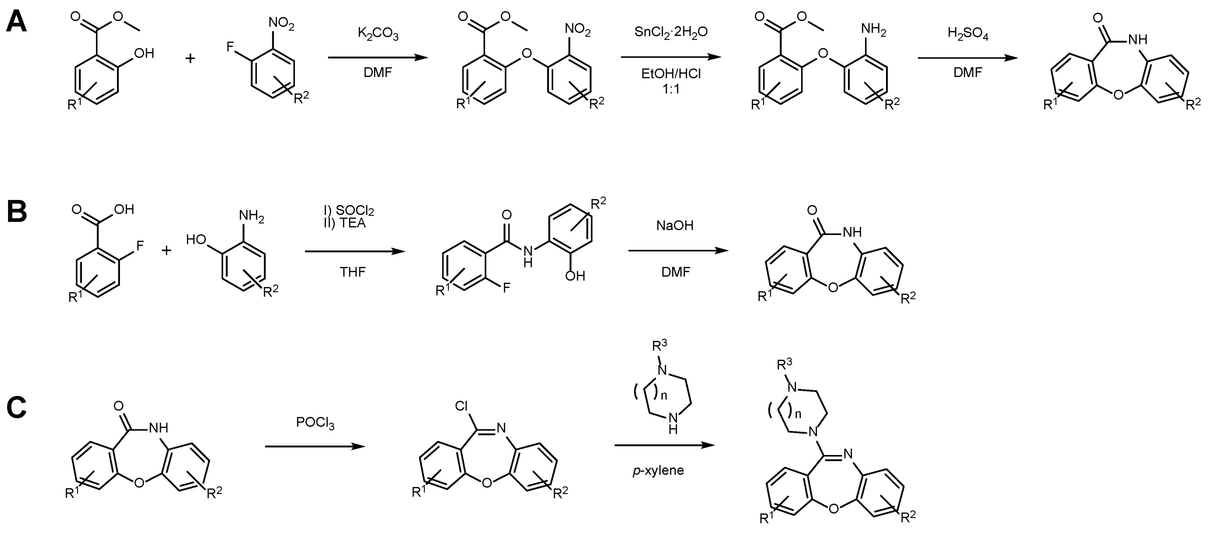

Figure S2. Chemical synthesis scheme of new compounds. A) General synthesis of lactam starting from methyl esters of salicylic acid and 2-fluoronitrobenzene derivatives. B) General synthesis of lactam starting from 2-fluorobenzoic acids and 2-aminophenol derivatives. C) General synthesis of loxapine derivatives starting from lactam.

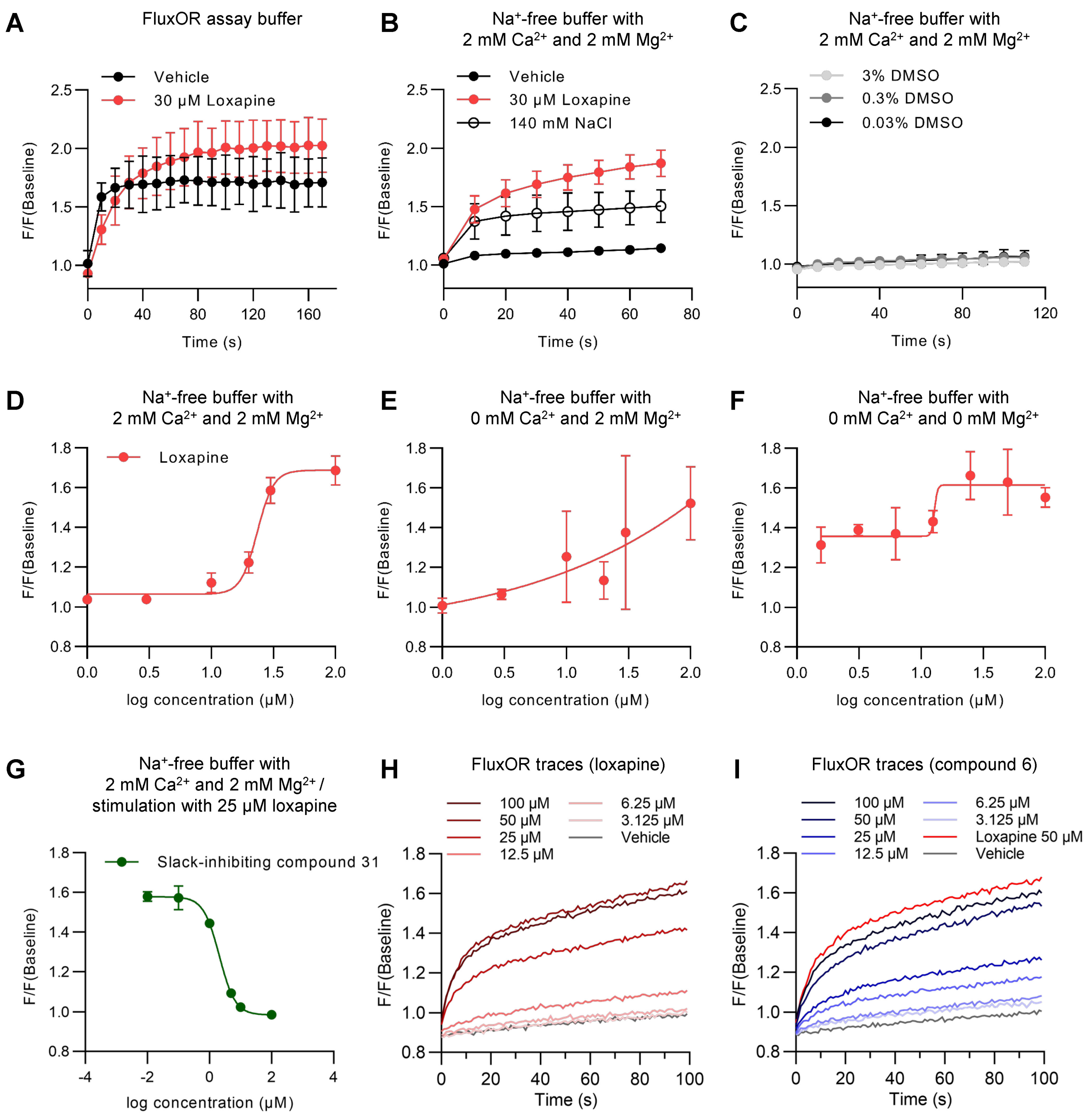

Figure S3. Establishing a modified version of the FluxOR assay. Cultured HEK293 cells stably expressing human Slack (HEK-Slack cells) were incubated with compounds in different buffers. A) In the buffer provided with the FluxOR assay kit, Slack activation (indicated as increased F/F(Baseline) ratio) was detected after incubation with both loxapine and vehicle (FluxOR assay buffer containing 0.03% DMSO). As Slack is activated by Na^+, the vehicle-induced Slack activation was most likely mediated by Na^+ present in the FluxOR assay buffer and in the vehicle. B) In a Na^+-free buffer (with replacement of NaCl by choline chloride), Slack activation was observed after incubation with 30 µM loxapine and 140 mM NaCl, but not after incubation with vehicle (Na^+-free buffer with 2 mM Ca^{2+}, 2 mM Mg^{2+} and 0.03% DMSO). C) DMSO at a concentration of 0.03-3% did not activate Slack in a Na^+-free

buffer with 2 mM Ca^{2+} and 2 mM Mg^{2+}. D) Concentration-response experiments with loxapine in a Na^{+}-free buffer with 2 mM Ca^{2+}, 2 mM Mg^{2+} and 0.03% DMSO yielded an EC_{50} value of 23.45 µM in the first preliminary experiments. E,F) As Slack is inhibited by bivalent cations, further control experiments with loxapine in a Na^{+} and Ca^{2+} free buffer (E) and in a Na^{+}, Ca^{2+} and Mg^{2+} free buffer (F) containing 0.03% DMSO were conducted, which however did not result in appropriate concentration-response curves. G) Concentration-response experiments with a Slack inhibitor (compound 31 from ref [5]) in a Na^{+}-free buffer with 2 mM Ca^{2+}, 2 mM Mg^{2+} and 0.03% DMSO and pre-stimulation with 25 µM loxapine revealed that compound 31 inhibited the F/F(Baseline) ratio in a concentration-dependent manner (IC_{50} = 2.1 µM), confirming that the readout depends on Slack. Therefore, all further experiments with the FluxOR assay in this study, which are presented in Figure 1A, were performed using a Na^{+}-free buffer with 2 mM Ca^{2+}, 2 mM Mg^{2+} and 0.03% DMSO. All conditions were measured at least in duplicate and data are shown as mean ± SD. H,I) Representative traces of (H) loxapine and (I) compound 6 from FluxOR experiments that are presented in Figure 1A. In (I), loxapine (50 µM) is included for better comparability.

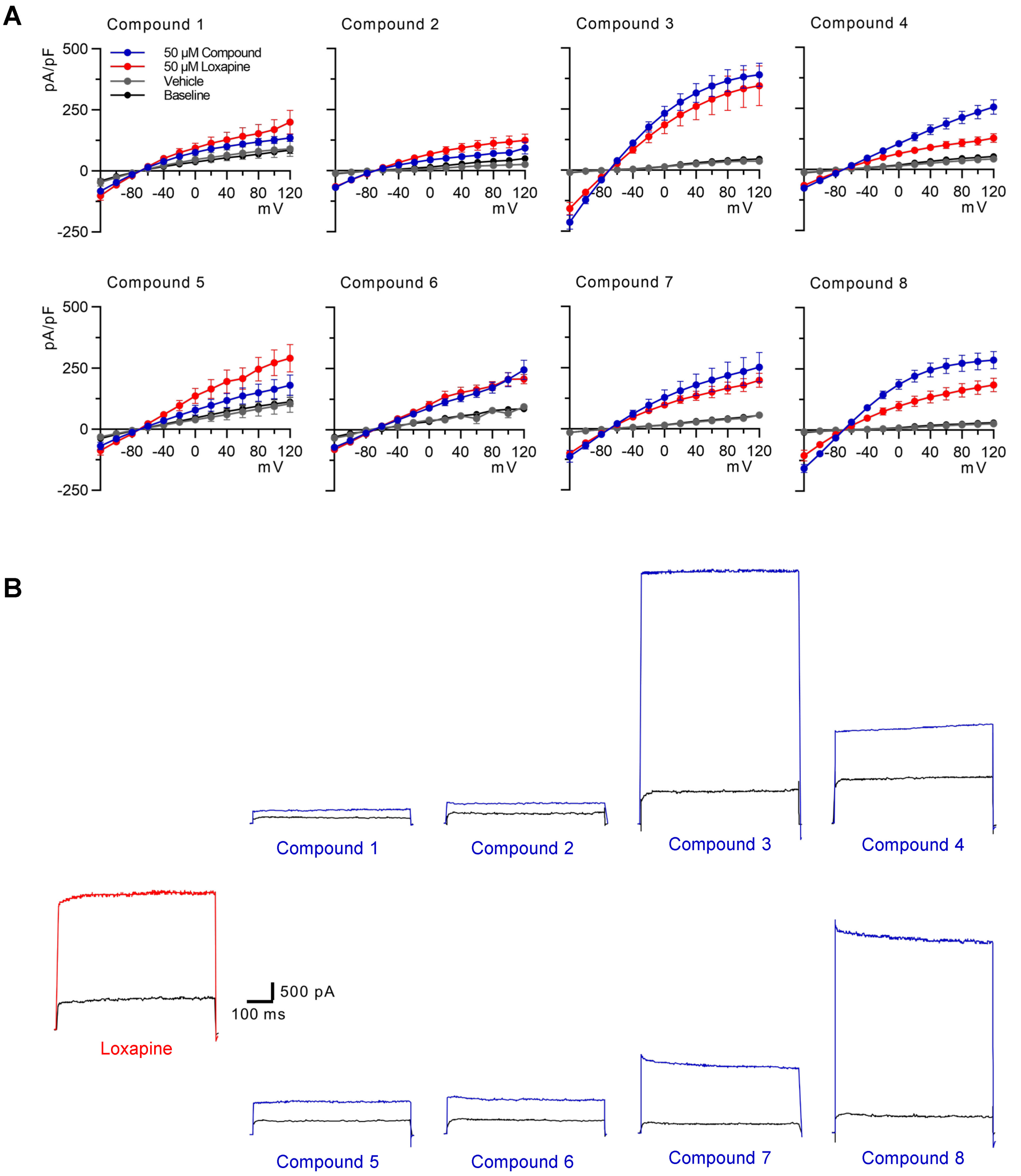

Figure S4. New compounds evoke Slack-mediated potassium currents. A) Current-voltage (I-V) curves from patch-clamp experiments that are presented in Figure 1B. Whole-cell voltage recordings on HEK-Slack cells were performed at baseline and after incubation with a new compound (50 µM), loxapine (50 µM) or vehicle (external solution containing 0.03% DMSO). Loxapine was incubated in each series of experiments as a positive control. *n* = 5-24 cells per group. Data are shown as mean ± SEM. B) Representative original current traces evoked by a voltage step to +80 mV recorded before (black) and after the application of loxapine (red) or the tested new compounds (blue). Data are from patch-clamp experiments pooled in Figure 1B.

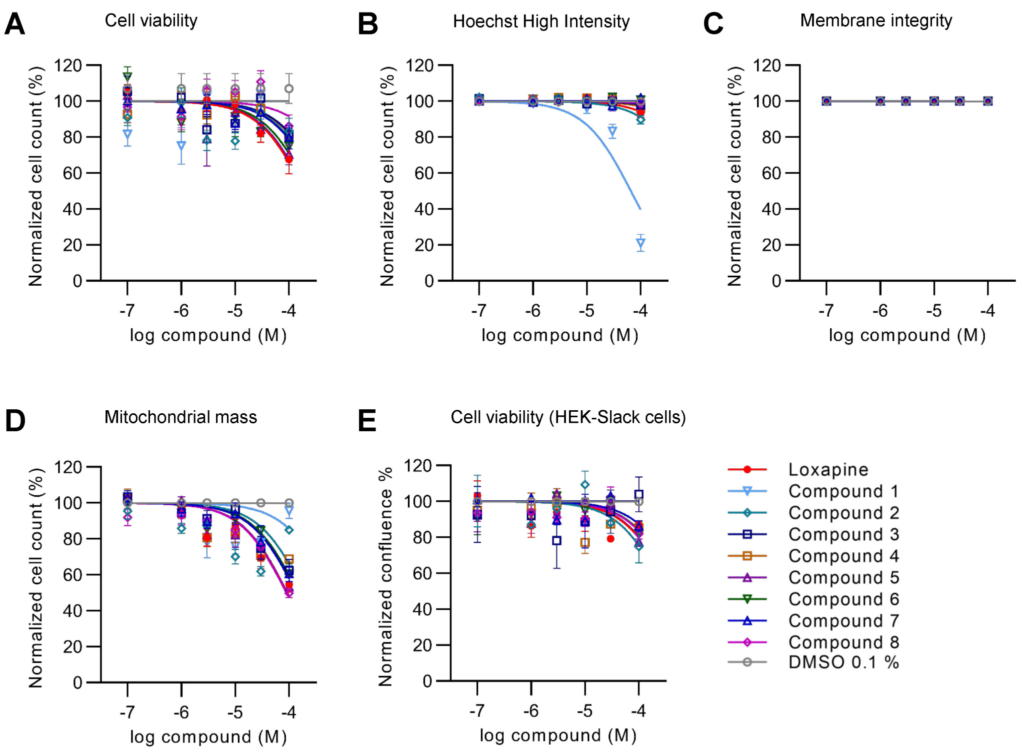

Figure S5. Cytotoxicity of new compounds. A–D) A live-cell phenotypic screening assay that allows for simultaneous investigation of cellular viability, nuclear morphology, mitochondrial mass and membrane integrity[6] was performed in HEK293 cells after 6 h incubation of new compounds at different concentrations. Cell counts were normalized against cells treated with 0.1 % DMSO (100%). A) None of the compounds decreased the cell viability by more than 50%, which is deemed a relevant impairment.[6] B) A decrease of normalized cell count to more than 50% based on Hoechst High Intensity objects, which indicates compound precipitation,[7] was only detected for compound 1 at 100 µM. C) No alterations in membrane integrity were detected. D) A decrease in mitochondrial mass by more than 50%, indicating cellular stress,[6] was only observed for compound 8 at 100 µM. E) In HEK-Slack cells, none of the compounds significantly impaired the confluence after 24 h incubation. Data are shown as mean ± SEM of biological duplicates.

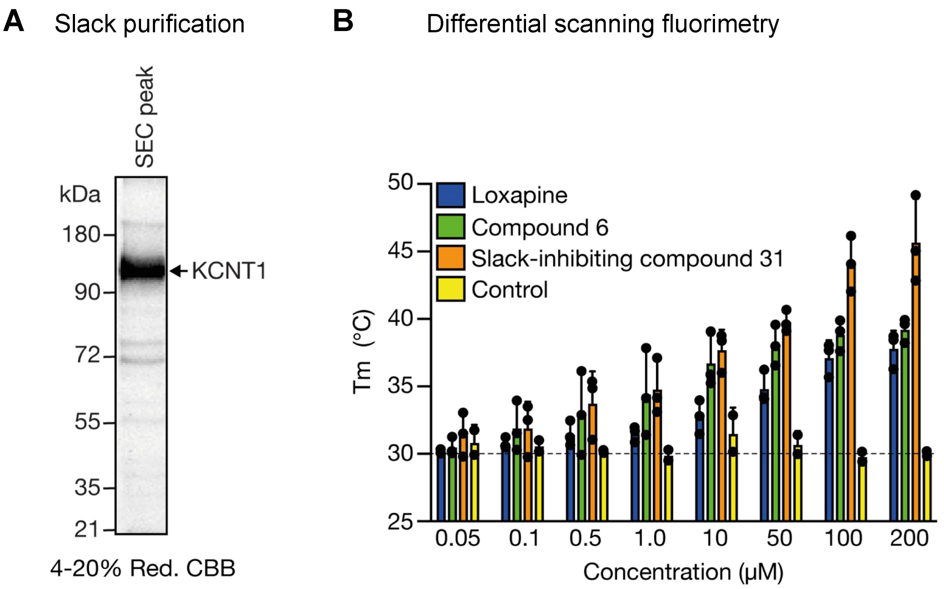

Figure S6. Target binding of compound 6 and loxapine. A) Slack was purified from HEK293F cells transiently expressing Slack via affinity purification and size-exclusion chromatography. B) Melting temperatures of purified Slack incubated with an increasing concentration of compound 6, loxapine and a Slack inhibitor (compound 31 from ref[5]) that was used as positive control. Purified Slack in buffer without any compound was used as a negative control. The melting temperature (T_m in °C) was determined with differential scanning fluorimetry (DSF) and plotted against the concentration of the compounds used. The dashed line indicates the T_m of purified Slack without compound. $n = 3$ experiments. Data are shown as mean ± SD.

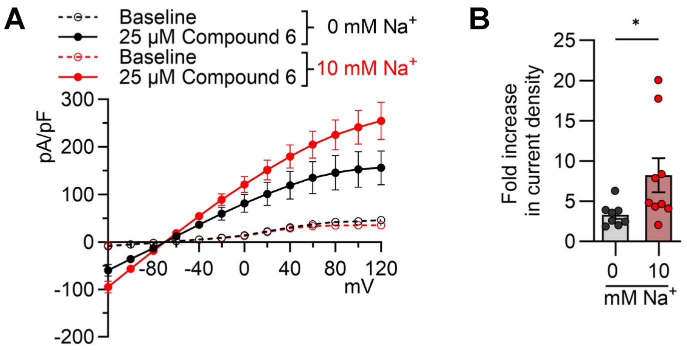

Figure S7. Slack-mediated potassium currents evoked by compound 6 are increased in presence of intracellular sodium. A) Current-voltage (I-V) curves from whole-cell voltage recordings on HEK-Slack cells at baseline and after incubation with compound 6 (25 µM). Recordings were performed with a Na^+-free pipette solution and with 10 mM Na^+ in the pipette solution. B) Fold increase of current densities shown in (A) relative to baseline at a voltage of +80 mV. Data show that compound 6 is more potent in the presence of intracellular Na^+. * $P < 0.05$, one-tailed unpaired Student's *t* test. n = 8–9 cells per group. Data are shown as mean ± SEM.

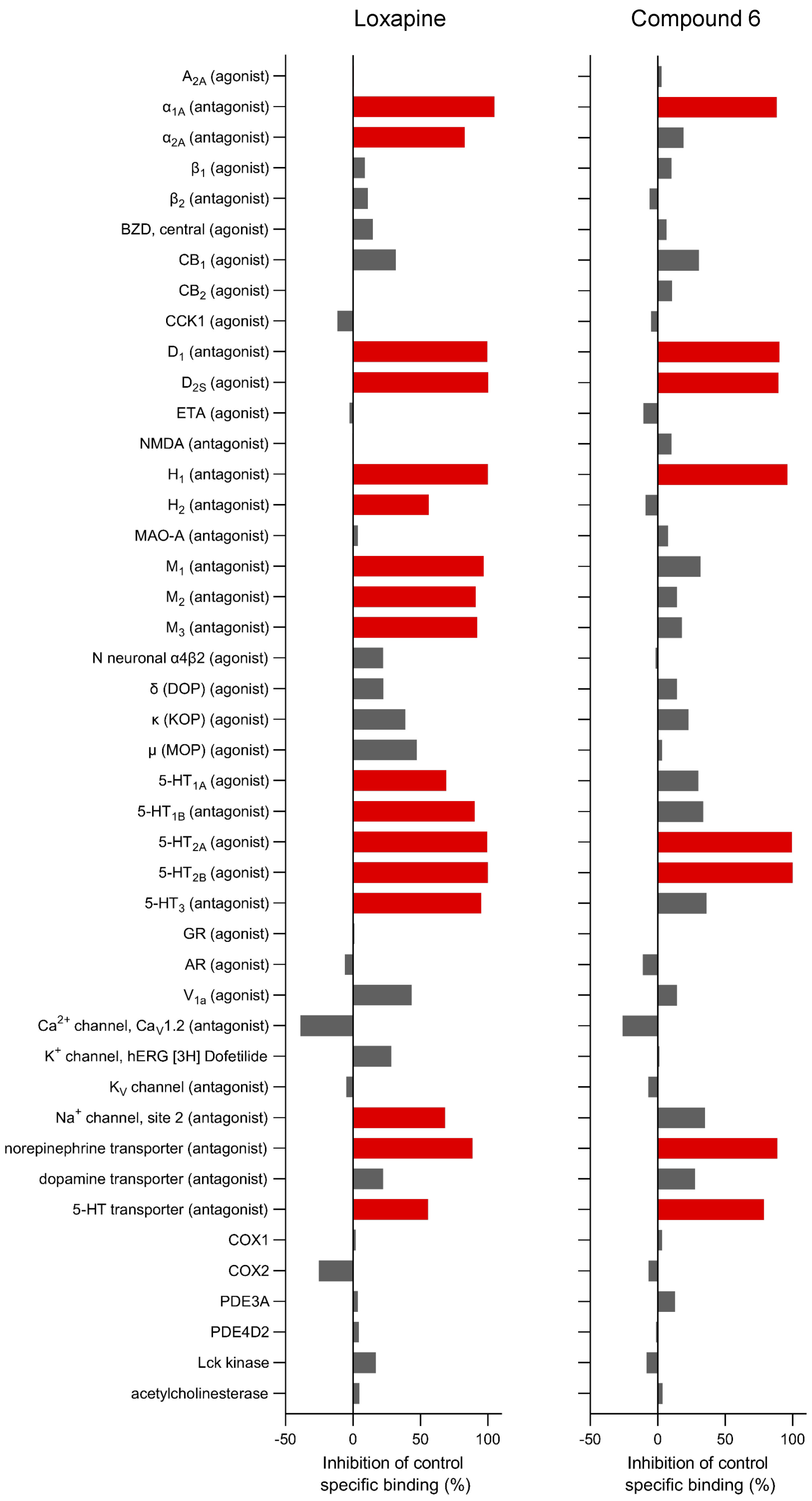

Figure S8. *In vitro* pharmacology screening of compound 6 and loxapine. Compounds were tested at a concentration of 10 μM in binding and enzyme and uptake assays of 44 targets (mostly human; except BZD, NMDA, MAO-A, Ca^{2+} channel, Kv channel, and Na^+

channel, which were obtained from rat specimens). Compound binding was calculated as a % inhibition of the binding of a radioactively labeled ligand (agonist or antagonist, as indicated in brackets) specific for each target. Compound enzyme inhibition effect was calculated as % inhibition of control enzyme activity. Results showing an inhibition (or stimulation for assays run in basal conditions) higher than 50% are considered to represent significant effects of the test compounds and are presented in red. Results showing an inhibition or stimulation between 25% and 50% (indicative of weak to moderate effects) and those lower than 25% (considered mostly attributable to variability of the signal around the control level) are presented in grey. Measurements were performed in duplicate.

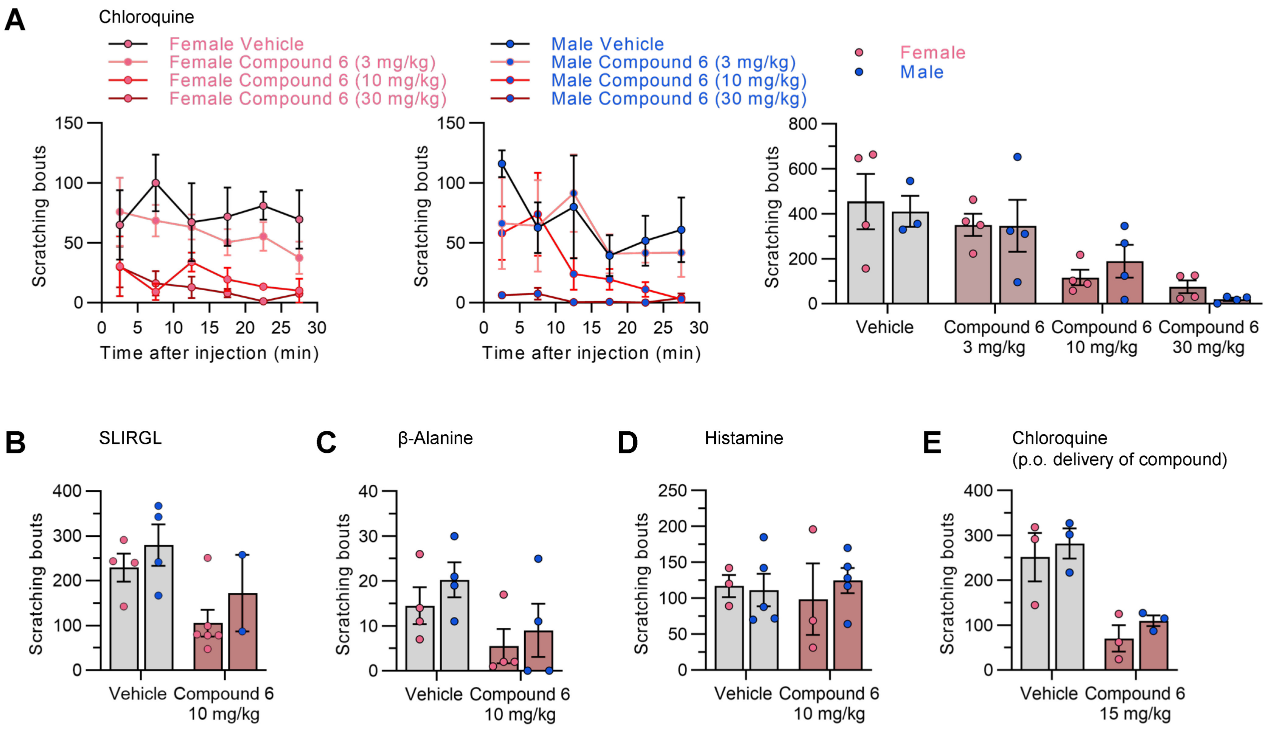

Figure S9. Sex-related effects of compound 6 in acute itch models. Breakdown of results in male and female mice from (A) Figure 3C, (B) Figure 3E, (C) Figure 3F, (D) Figure 3G, and (E) Figure 3H. No obvious sex-related differences were observed in any assay. Statistical significance was assessed by one-way-ANOVA with Dunnett's correction. Data represent the mean ± SEM (n = 2–5).

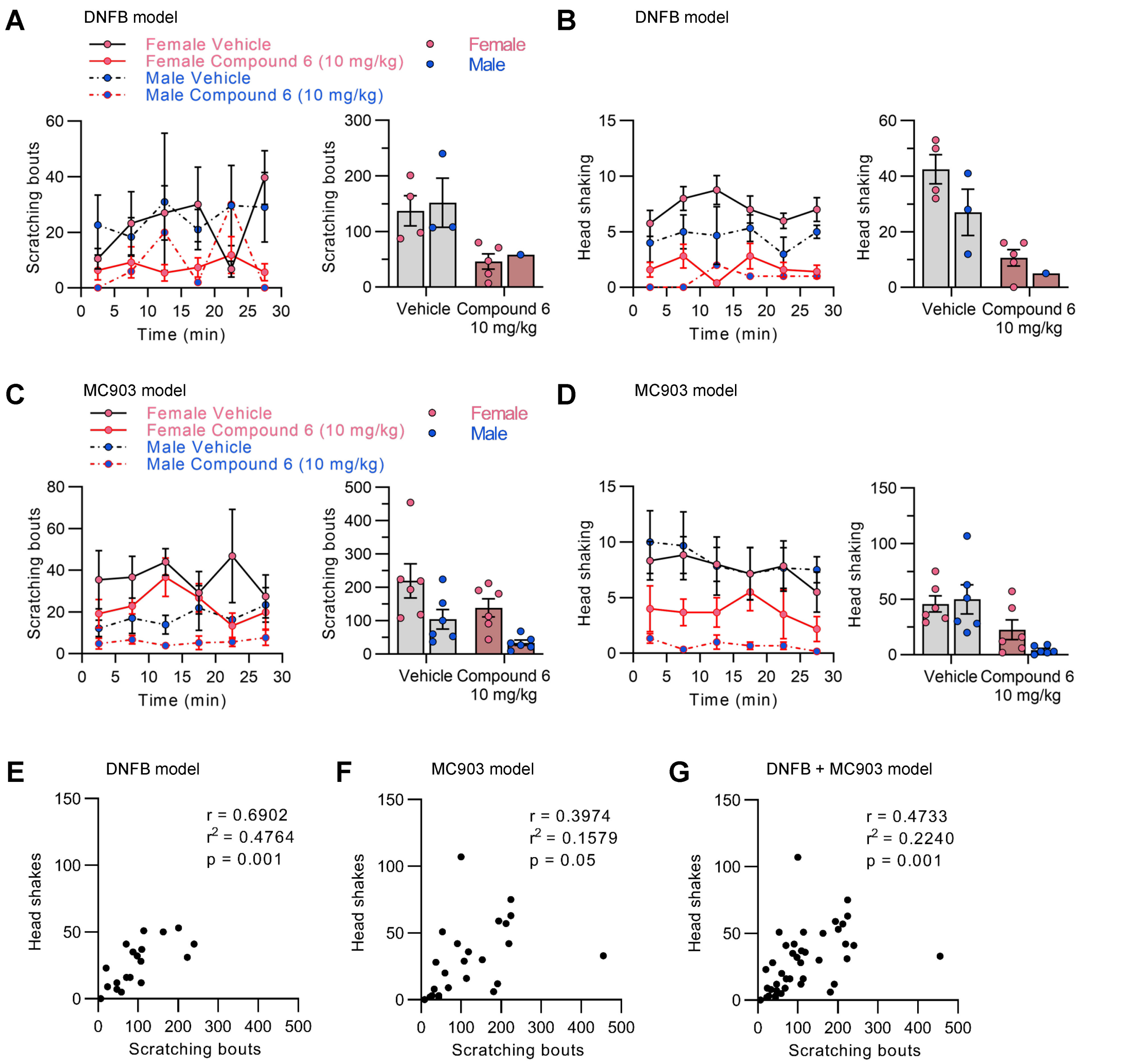

Figure S10. Sex-related effects of compound 6 and correlation of behavioral outcomes in chronic itch models. A-D) Breakdown of results in male and female mice from (A) Figure 4B, (B) Figure 4C, (C) Figure 4E, and (D) Figure 4F. There was a tendency toward reduced chronic itch behavior in males compared to females (head shaking in the DNFB model and scratching in the MC903 model), albeit not significant. Statistical significance was assessed by one-way-ANOVA with Dunnett's correction. Data represent the mean ± SEM (n = 1–6). E–G) Correlation of number of head shakes with number of scratching bouts (E) in the DNFB model (Figure 4B,C), (F) in the MC903 model (Figure 4E,F) and (G) in both models. Statistical significance was assessed by a Pearson correlation.

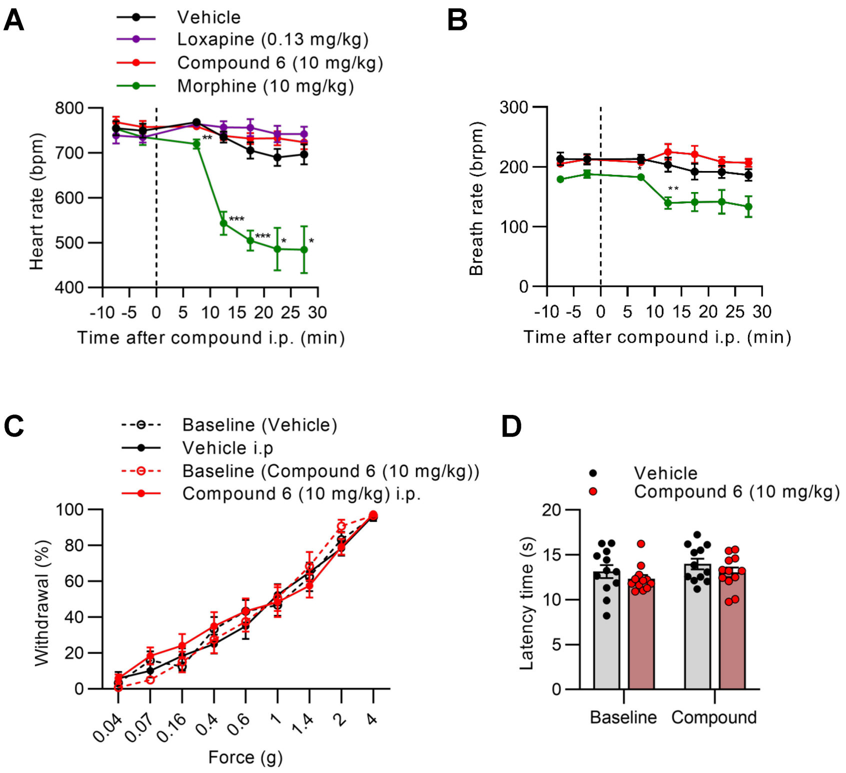

Figure S11. Potential side effects of compound 6. A,B) Pulse oximetry on non-anesthetized mice using a MouseOX Plus device revealed that i.p. delivery of compound 6 or vehicle did not alter (A) the heart rate or (B) the breath rate, whereas morphine significantly lowered both parameters ($n = 6$; *$P < 0.05$, **$P < 0.01$, ***$P < 0.001$ vs vehicle, two-way MC ANOVA and Dunnett test). C,D) Sensing of (C) mechanical stimuli (von Frey filament test) and (D) heat stimuli (Hargreaves test) was not affected after i.p. delivery of compound 6 or vehicle ($n = 12$). Data represent the mean ± SEM.

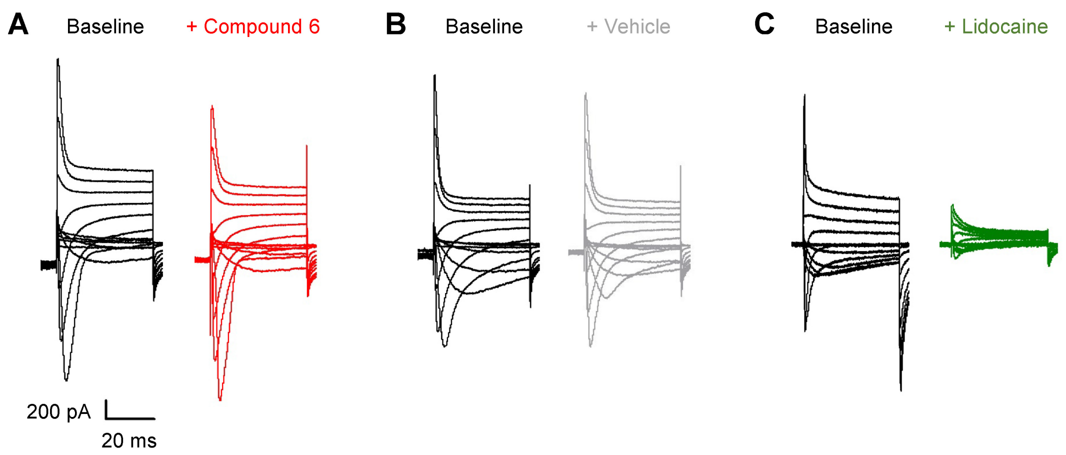

Figure S12. Tetrodotoxin-resistant Na^+ currents in IB4-binding DRG neurons are not affected by compound 6. Representative recordings of whole-cell I_{Na} superfused with 250 nM tetrodotoxin under control conditions (black) and after addition of (A) 50 µM compound 6 (red), (B) vehicle (external solution containing 0.03% DMSO; grey), or (C) 50 µM lidocaine (green), which was used as a positive control. I_{Na} was elicited after a 700 ms prepulse in 10 mV depolarizing steps from -80 mV to +40 mV for 50 ms. Recordings were performed in 3 IB4-binding DRG neurons per group.

	D_2/D_3 receptor affinity	
**Compounds**	D_2 receptor	D_3 receptor
	K_i (nM) [CI 95%] (n)	
Compound 6	1281 [888 – 1848] (3)	3520 [2485 – 4986] (3)
Compound 8	134 [79.6 – 226] (4)	421 [212 – 833] (4)
Compound 2	82.8 [37.1 – 185] (5)	62.4 [22.6 – 172] (4)
Compound 4	50 [31 – 80] (3)	176 [159 – 194] (3)
Compound 5	44.9 [13.4 – 150] (4)	63.4 [52.7 – 76.2] (3)
Loxapine	32 [10 – 103] (4)	112 [92 – 137] (4)
Compound 7	27.4 [11.4 – 66.2] (4)	40.7 [6.62 – 250] (4)
Compound 1	3.6 [1.06 – 12.2] (4)	12.2 [3.71 – 40] (4)
Compound 3	2.89 [1.09 – 7.62] (4)	22.6 [3.49 – 146] (4)

Table S1. Dopamine D_2/D_3 receptor affinity of the new compounds. D_2/D_3 receptor affinity values are from data presented in Figure 2B. [^{3}H]-Spiperone competition binding assays were performed using a cell membrane preparation of CHO cells stably expressing the human D_{2s} and D_3 receptor. K_i data represent mean with the 95% confidence interval (CI 95%) of *n* independent experiments, each performed in triplicate using seven appropriate concentrations of test compound. Haloperidol was used as a reference compound and to determine non-specific binding at 10 μM.

Compound	Route (dose)	PK parameters	Plasma	Brain
Compound 6	I.P. (10 mg/kg)	C_{max} (ng/mL)<br>t_{max} (min)<br>AUC_{0-inf} (ng*min/mL)<br>$t_{1/2}$ (min)	6280<br>15<br>336000<br>35.0	5490<br>15<br>407000<br>39.7
	P.O. (10 mg/kg)	C_{max} (ng/mL)<br>t_{max} (min)<br>AUC_{0-inf} (ng*min/mL)<br>$t_{1/2}$ (min)	3140<br>15<br>244000<br>67.4	2460<br>15<br>225000<br>71.9
	I.V. (1 mg/kg)	C_{max} (ng/mL)<br>t_{max} (min)<br>AUC_{0-inf} (ng*min/mL)<br>$t_{1/2}$ (min)	562<br>-<br>28400<br>52.5	493<br>15<br>31200<br>62.1
Loxapine	I.P. (2.5 mg/kg)	C_{max} (ng/mL)<br>t_{max} (min)<br>AUC_{0-inf} (ng*min/mL)<br>$t_{1/2}$ (min)	143<br>15<br>10700<br>61.9	493<br>15<br>54800<br>82.2

Table S2. Pharmacokinetic parameters of compound 6 and loxapine in mice. Animals were i.p., p.o. or i.v. injected with the compound at the indicated dose and plasma or brain concentration at different time points (0.25, 0.5, 1, 2 and 4 h after i.p. delivery; 0.25, 0.5, 1, 2, 4 and 8 h after p.o. delivery; 0.083, 0.25, 0.5, 1, 2 and 4 h after i.v. delivery) were measured by LC-MS analysis (*n* = 3 mice per group). C_{max}: maximum concentration reached in plasma or brain; t_{max}: time to reach C_{max}; AUC_{0-inf}: area under the concentration-time curve from zero to time infinity; $t_{1/2}$: half-life.

Compound	K_i (nM) [CI 95%] (n)
Chlorphenamine maleate	9.07 [1.98 – 41.7] (5)
Loxapine	2.72 [0.65 – 11.5] (5)
Compound 6	575 [137 – 2422] (5)

Table S3. Histamine H_1 receptor affinity of compound 6 and loxapine. [^{3}H]-Pyrilamine competition binding assays were performed using cell membrane preparation of CHO cells stably expressing the human H_1 receptor. K_i data represent mean with the 95% confidence interval (CI 95%) of *n* independent experiments, each performed in triplicate using seven appropriate concentrations of test compound. Chlorphenamine maleate was used as reference compound. Non-specific binding was determined in the presence of 10 µM chlorphenamine maleate.

Supplemental protocols for synthesis of compounds

(A) General synthesis of lactam starting from methyl esters of salicylic acid and 2-fluoronitrobenzene derivatives.

GP1: Formation of the diarylether

Potassium carbonate (1.5 equiv) was added to a solution of the corresponding methyl salicylate (1.5 equiv) and the corresponding 2-fluoronitrobenzene (1.0 equiv) in DMF (1.5 M regarding 2-fluoronitrobenzene). The resulting solution was heated in an oil bath to 120 °C overnight. Solvents were evaporated under reduced pressure and the residue taken into water and extracted three times with ethyl acetate. The combined organic phases were dried over magnesium sulfate, filtered, and evaporated. The resulting crude product was purified by flash chromatography.

GP2: Reduction of the nitro group

A solution of $SnCl_2 \cdot 2H_2O$ (4.0 equiv) in conc. HCl (3 M) was added to a solution of the corresponding methyl 2-(2-nitrophenoxy)benzoate derivative (1.0 equiv) in a mixture of ethanol/conc. HCl 1:1 (0.5 M). The resulting solution was stirred at rt overnight. After that time, the temperature was set to 0 °C and the pH of the reaction solution made slightly basic by addition of sodium carbonate. The resulting solution was then extracted three times with ethyl acetate. The combined organic phases were dried over magnesium sulfate, filtered, and evaporated. The resulting crude product was purified by flash chromatography.

GP3: Formation of the lactam ring via intramolecular condensation

A solution of the corresponding methyl 2-(2-aminophenoxy) benzoate derivative (1.0 equiv) in DMF (0.2 M) was treated with concentrated sulfuric acid (1.3 equiv) and heated in an oil bath at 120 °C overnight. After that time, the reaction was cooled to 0 °C and a few millilitres of water were added. The precipitated product was then filtered off and dried under vacuum to obtain the crude product, which was used in the next step without further purification.

(B) General synthesis of lactam starting from 2-fluorobenzoic acids and 2-aminophenol derivatives.

GP4: Formation of the amide

To a solution of the corresponding 2-fluorobenzoic acid (1.0 equiv) in THF (1 M) freshly distilled thionylchloride (2.0 equiv) was added and heated to reflux in an oil bath for 2 h. Thereafter, excess thionylchloride and THF were removed under reduced pressure and the residue obtained was taken up in THF (2.5 M) again. This solution was added dropwise to a solution of the corresponding 2-aminophenol derivative (1.0 equiv) and triethylamine (2 equiv) in THF (2.5 M referred to 2-aminophenol derivative) at 0 °C and the reaction mixture was stirred overnight at rt. After that time, the reaction mixture was concentrated under reduced pressure and the residue taken up in ethyl acetate. The organic phase was first washed with an aq HCl solution (2 M), water and saturated aq NaCl solution, then dried over magnesium sulfate, filtered, and evaporated. The resulting crude product was purified by flash chromatography.

GP5: Formation of the lactam ring via intramolecular nucleophilic aromatic substitution

A solution of the corresponding 2-fluoro-*N*-(2-hydroxyphenyl)benzamide derivative (1.0 equiv) in DMF (0.25 M) was treated with freshly powdered NaOH (1.0 equiv) and heated in an oil bath at 150 °C for 5 h. After that time, the reaction was cooled to 0 °C and a few millilitres of water were added. The precipitated product was then filtered off and dried under vacuum to obtain the crude product, which was used in the next step without further purification.

(C) General synthesis of loxapine derivatives starting from lactam

GP6: Reaction with phosphorus oxychloride

The corresponding lactam (1.0 equiv) was dissolved in freshly distilled phosphorus oxychloride (0.5 M) and *N*,*N*-dimethylaniline (0.6 equiv) was added. The resulting mixture was heated to reflux in an oil bath for 5 h. Thereafter, excess phosphorus oxychloride was removed under reduced pressure and the residue obtained was taken up in toluene and washed once with cold water. The organic phase was dried over magnesium sulfate, filtered, and evaporated to give the crude product, which was immediately used in the next step without further purification.

GP7: Formation of loxapine derivatives

The corresponding amine (2.0 equiv) was added to a solution of the appropriate imidoylchloride (1.0 equiv) in *p*-xylene (0.15 M regarding imidoylchloride). The resulting

mixture was heated in an oil bath at 140 °C for 5 h. Solvents were evaporated, and the crude substance purified by preparative HPLC or flash chromatography. If unsubstituted piperazine or homopiperazine was employed, then alkylation of these derivatives was obtained by GP8

GP8: Alkylation of loxapine derivatives

A mixture of loxapine derivative (1 equiv), corresponding alkyl chloride (2 equiv) and triethylamine (10 equiv) in acetonitrile (0.15 M) was heated to reflux in an oil bath for 16 h. After completion, the reaction mixture was evaporated, and the residue purified by preparative HPLC or flash chromatography.

Characterization of compounds

Methyl 2-methoxy-5-(trifluoromethyl)benzoate (9)

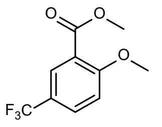

A solution of 2-methoxy-5-(trifluoromethyl)benzoic acid (1.00 g, 4.41 mmol) in MeOH (10 mL) was treated with concentrated sulfuric acid (235 µL, 4.41 mmol) and heated to reflux in an oil bath overnight. The reaction mixture was concentrated under reduced pressure and the residue was taken up in water. The aqueous phase was extracted three times with ethyl acetate and the combined organic phases were dried over magnesium sulfate and filtered. Concentrating the reaction mixture under reduced pressure yielded the title compound as a colourless oil (1.02 g, quantitative). ^{1}H NMR (250 MHz, acetone-d_6) δ 7.99 (d, J = 2.4 Hz, 1H), 7.86 (dd, J = 8.8, 2.4 Hz, 1H), 7.36 (d, J = 8.8 Hz, 1H), 3.98 (s, 3H), 3.86 (s, 3H).

Methyl 2-hydroxy-5-(trifluoromethyl)benzoate (10)

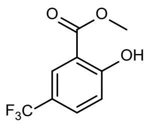

A solution of boron tribromide (7.8 mL, 7.80 mmol) in DCM (1 M) was added to a solution of **9** (939 mg, 3.89 mmol) in DCM (30 mL) at -78 °C and the resulting mixture was first stirred at -78 °C for 5 min, then at 0 °C for 10 min. A few mL of MeOH were added before the reaction mixture was concentrated under reduced pressure and the residue was diluted

with ethyl acetate. The organic phase was washed twice with water and once with a saturated aq NaCl solution, dried over magnesium sulfate, and filtered. Concentrating the reaction mixture under reduced pressure yielded the title compound as a brown oil (364 mg, 43%). ^{1}H NMR (250 MHz, acetone-*d*$_6$) δ 11.11 (s, 1H), 8.15 (d, J = 1.9 Hz, 1H), 7.86 (dd, J = 8.8, 2.4 Hz, 1H), 7.18 (d, J = 8.8 Hz, 1H), 4.03 (s, 3H).

Methyl 5-chloro-2-(2-nitrophenoxy)benzoate (**11**)

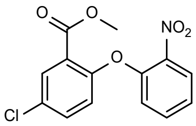

Synthesized according to GP1 from methyl 5-chlorosalicylate (2.80 g, 15.0 mmol), 2-fluoronitrobenzene (1.06 mL, 10.0 mmol) and potassium carbonate (2.07 g, 15 mmol). Purification of the crude product by flash chromatography (*n*-hexane/EtOAc 9:1 to 8:2) yielded the title compound as a colourless solid (3.07 g, 99%). ^{1}H NMR (250 MHz, DMSO-*d*$_6$) δ 8.07 (dd, *J* = 8.1, 1.7 Hz, 1H) 7.92 (d, *J* = 2.7 Hz, 1H), 7.75 (dd, *J* = 8.8, 2.8 Hz, 1H) 7.68-7.60 (m, 1H), 7.37-7.30 (m, 1H), 7.27 (d, *J* = 8.8 Hz, 1H), 7.00 (d, *J* = 8.4, 1.1 Hz, 1H), 3.70 (s, 3H).

Methyl 2-(2-nitrophenoxy)-5-(trifluoromethyl)benzoate (**12**)

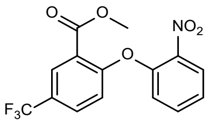

Synthesized according to GP1 from **10** (991 mg, 4.50 mmol), 2-fluoronitrobenzene (317 µL, 3.00 mmol) and potassium carbonate (622 mg, 4.50 mmol). Purification of the crude product by flash chromatography (hexane/EtOAc 9:1 to 8:2) yielded the title compound as a colourless solid (615 mg, 60%). ^{1}H NMR (250 MHz, $CDCl_3$) δ 8.27 (s, 1H) 8.03 (d, *J* = 8.2 Hz, 1H), 7.75 (d, *J* = 8.6 Hz, 1H), 7.56 (t, *J* = 8.2 Hz, 1H), 7.29 (t, *J* = 8.2 Hz, 1H), 7.08 (d, *J* = 8.6 Hz, 1H), 6.98 (d, *J* = 8.3 Hz, 1H), 3.85 (s, 3H).

Methyl 2-(2-aminophenoxy)-5-chlorobenzoate (**13**)

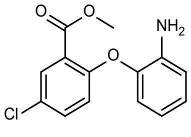

Synthesized according to GP2 from **11** (954 mg, 3.10 mmol) and tin (II) chloride dihydrate (2.80 g, 12.4 mmol). Purification of the crude product by flash chromatography (hexane/EtOAc 9:1 to 8:2) yielded the title compound as a colourless solid (635 mg, 74%). ^{1}H NMR (300 MHz, $CDCl_3$) δ 7.82 (d, J = 2.6 Hz, 1H), 7.33 (dd, J = 8.7, 2.6 Hz, 1H), 7.04-6.97 (m, 1H), 6.88-6.80 (m, 3H), 6.71 (td, J = 7.7, 1.5 Hz, 1H), 3.89 (s, 3H), 3.64 (br s, 2H).

Methyl 2-(2-aminophenoxy)-5-(trifluoromethyl)benzoate (14)

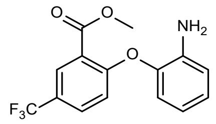

Synthesized according to GP2 from **12** (334 mg, 0.980 mmol) and tin (II) chloride dihydrate (900 mg, 3.91 mmol). Purification of the crude product by flash chromatography (hexane/EtOAc 9:1 to 8:2) yielded the title compound as a colourless solid (171 mg, 56%). ^{1}H NMR (250 MHz, $CDCl_3$) δ 8.12 (d, J = 2.1 Hz, 1H), 7.61 (dd, J = 8.7, 2.4 Hz, 1H), 7.10-7.02 (m, 1H), 6.98-6.93 (m, 2H), 6.84 (dd, J = 7.9, 1.5 Hz, 1H), 6.79-6.72 (m, 1H), 3.99 (br s, 2H), 3.94 (s, 3H).

2-Chlorodibenzo[*b*,*f*][1,4]oxazepin-11(10*H*)-one (15)

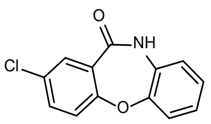

Synthesized according to GP3 from **13** (630 mg, 2.27 mmol) and concentrated sulphuric acid (150 µL, 2.81 mmol). After filtration the title compound was obtained as a colourless solid (508 mg, 90%). ^{1}H NMR (250 MHz, DMSO-d_6) δ 10.66 (s, 1H), 7.72 (d, J = 2.5 Hz, 1H) 7.67 (dd, J = 8.6, 2.8 Hz, 1H), 7.40 (d, J = 8.6 Hz, 1H) 7.34 (dt, J = 7.1, 1.2 Hz, 1H), 7.21-7.10 (m, 3H).

2-(Trifluoromethyl)dibenzo[*b*,*f*][1,4]oxazepin-11(10*H*)-one (16)

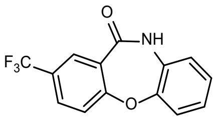

Synthesized according to GP3 from **14** (166 mg, 0.533 mmol) and concentrated sulphuric acid (35.3 µL, 0.663 mmol). After filtration the title compound was obtained as a colourless solid

(73 mg, 49%). ^{1}H NMR (250 MHz, DMSO-d_6) δ 10.77 (s, 1H), 8.05-7.98 (m, 2H) 7.59 (d, J = 8.3 Hz, 1H), 7.39 (dt, J = 7.3, 1.2 Hz, 1H), 7.23-7.13 (m, 3H).

2-Fluoro-*N*-(5-fluoro-2-hydroxyphenyl)-5-(trifluoromethyl)benzamide (17)

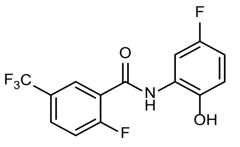

Synthesized according to GP4 from 2-fluoro-5-(trifluoromethyl)benzoic acid (1.50 g, 7.06 mmol), thionylchloride (1.04 mL, 14.1 mmol), 2-amino-4-fluorophenol (925 mg, 7.06 mmol) and triethylamine (1.98 mL, 14.1 mmol). Purification of the crude product by flash chromatography (*n*-hexane/EtOAc 99:1 to 2:3) yielded the title compound as a red solid (1.43 g, 64%). ^{1}H NMR (400 MHz, DMSO-d_6) δ 8.17-8.13 (m, 1H), 8.05-7.91 (m, 2H), 7.66-7.59 (m, 1H), 6.93-6.80 (m, 2H).

8-Fluoro-2-(trifluoromethyl)dibenzo[*b*,*f*][1,4]oxazepin-11(10*H*)-one (18)

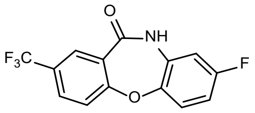

Synthesized according to GP5 from **17** (196 mg, 0.618 mmol), and sodium hydroxide (25 mg, 0.618 mmol). After filtration the title compound was obtained as a light-brown solid (168 mg, 92%). ^{1}H NMR (250 MHz, DMSO-d_6) δ 10.85 (s, 1H), 8.04-8.00 (m, 2H), 7.61-7.58 (m, 1H), 7.48-7.40 (m, 1H), 7.06-6.97 (m, 2H).

2,11-Dichlorodibenzo[*b*,*f*][1,4]oxazepine (19)

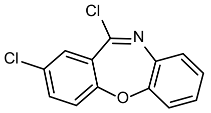

Synthesized according to GP6 from **15** (558 mg, 2.27 mmol), phosphorus oxychloride and *N*,*N*-dimethylaniline (173 µL, 1.36 mmol). After work up the title compound was obtained as brown solid (598 mg, 99%).

11-Chloro-2-(trifluoromethyl)dibenzo[*b*,*f*][1,4]oxazepine (20)

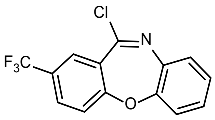

Synthesized according to GP6 from **16** (73 mg, 0.261 mmol), phosphorus oxychloride and *N*,*N*-dimethylaniline (20.3 µL, 0.160 mmol). After work up the title compound was obtained as a brown solid (64 mg, 82%).

2,11-Dichlorodibenzo[*b*,*f*][1,4]thiazepine (21)

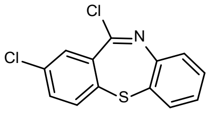

Synthetized according to GP6 from commercially purchased 2-chlorodibenzo[*b*,*f*][1,4]thiazepin-11(10*H*)-one (BLDPharm, Cat. No.: BD223305 97%) (125 mg, 0.478 mmol), phosphorus oxychloride and *N*,*N*-dimethylaniline (36.8 µL, 0.290 mmol). After work up the title compound was obtained as a brown solid (124 mg, 92%).)

11-Chloro-8-fluoro-2-(trifluoromethyl)dibenzo[*b*,*f*][1,4]oxazepine (22)

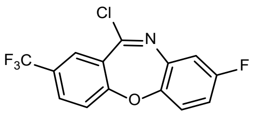

Synthetized according to GP6 from **18** (120 mg, 0.404 mmol), phosphorus oxychloride and *N*,*N*-dimethylaniline (31.0 µL, 0.242 mmol). After work up the title compound was obtained as a light-brown solid. (50.4 mg, 39%).

11-(Piperazin-1-yl)-2-(trifluoromethyl)dibenzo[*b*,*f*][1,4]oxazepine (23)

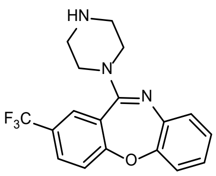

Synthesized according to GP7 from **20** (267 mg, 0.897 mmol) and piperazine (155 mg, 1.79 mmol) in. Purification of the crude product by flash chromatography (DCM/MeOH 95:5 to 9:1) yielded the title compound as a yellow solid (240 mg, 77%). ^{1}H NMR (400 MHz, $CDCl_3$) δ 8.77 (s, 2H), 8.47 (br s, 1H), 7.73 (d, *J* = 8.0 Hz, 1H), 7.64-7.60 (m, 1H), 7.37 (d, *J* = 8.0 Hz, 1H), 7.18-7.03 (m, 4H), 3.77-3.23 (m, 8H).

11-(1,4-Diazepan-1-yl)-2-(trifluoromethyl)dibenzo[*b*,*f*][1,4]oxazepine (24)

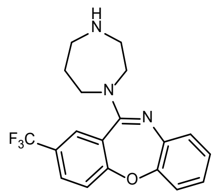

Synthesized according to GP7 from **20** (267 mg, 0.897 mmol) and homopiperazine (183 mg, 1.79 mmol). Purification of the crude product by flash chromatography (DCM/MeOH 95:5 to 9:1) yielded the title compound as a yellow solid (178 mg, 55%). ^{1}H NMR (400 MHz, $CDCl_3$) δ 7.69 (dd, *J* = 8.6, 2.0, 1H), 7.63 (d, *J* = 1.9 Hz, 1H), 7.36 (d, *J* = 8.5 Hz, 1H), 7.13-7.06 (m, 3H), 7.99-6.95 (m, 1H), 3.98-3.13 (m, 9H), 2.14-1.90 (m, 2H).

11-(4-Methylpiperazin-1-yl)-2-(trifluoromethyl)dibenzo[*b*,*f*][1,4]oxazepine (**1**)

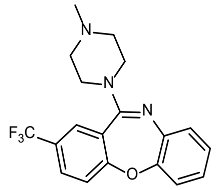

Synthesized according to GP7 from **20** (63 mg, 0.210 mmol) and 1-methylpiperazine (46.6 µL, 0.420 mmol). Purification of the crude product by preparative HPLC yielded the title compound as a brown oil and formate salt (22 mg, 26%). ^{1}H NMR (300 MHz, $CDCl_3$) δ 8.35 (s, 1H), 7.95 (br s, 1H), 7.70 (ddd, *J* = 8.5, 2.3, 0.5 Hz, 1H), 7.62 (d, *J* = 2.3 Hz, 1H), 7.36 (d, *J* = 8.5 Hz, 1H), 7.18-7.07 (m, 3H), 7.01 (td, *J* = 7.4, 2.0 Hz, 1H), 3.62 (br s, 4H), 2.69 (br s, 4H), 2.44 (s, 3H); $^{13}C\{^{1}H\}$ NMR (75 MHz, $CDCl_3$) δ 166.6, 163.2, 158.7, 151.4, 139.9, 129.7, 127.5, 127.2, 127.0, 126.0, 124.9, 124.1, 123.5, 122.1, 120.3, 54.0, 46.7, 45.3; HPLC-purity (254 nm): 96%; MALDI-HRMS: *m/z* calculated for $C_{19}H_{19}F_3N_3O[M+H]^+$: 362.1474, found: 362.1488.

11-(4-Methyl-1,4-diazepan-1-yl)-2-(trifluoromethyl)dibenzo[*b*,*f*][1,4]oxazepine (**2**)

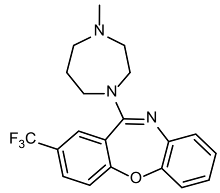

Synthesized according to GP7 from **20** (63 mg, 0.212 mmol) and 1-methylhomopiperazine (49 mg, 0.423 mmol). Purification of the crude product by preparative HPLC yielded the title compound as a brown oil and formate salt (73 mg, 92%). ^{1}H NMR (300 MHz, $CDCl_3$) δ 9.97

(br s, 1H), 8.51 (s, 1H), 7.68 (dd, *J* = 8.5, 1.9 Hz, 1H), 7.58 (d, *J* = 2.1 Hz, 1H), 7.35 (d, *J* = 8.5 Hz, 1H), 7.12-7.06 (m, 3H), 6.98-6.95 (m, 1H), 4.06-3.45 (m, 4H), 3.08-2.84 (m, 4H), 2.56 (s, 3H), 2.29 (br s, 1H), 2.01 (br s, 1H); $^{13}C\{^1H\}$ NMR (75 MHz, $CDCl_3$) δ 168.2, 162,8, 158.3, 151.2, 140.4, 129.4, 127.4, 127.0, 126.9, 126.2, 124.2, 124.0, 123.6, 122.2, 120.2, 56.9, 56.3, 49.1, 46.9, 45.4, 26.3; HPLC-purity (254 nm): 97%; MALDI-HRMS: *m/z* calculated for $C_{20}H_{21}F_3N_3O[M+H]^+$: 376.1631, found: 376.1639.

8-Fluoro-11-(4-methylpiperazin-1-yl)-2-(trifluoromethyl)dibenzo[*b*,*f*][1,4]oxazepine (**3**)

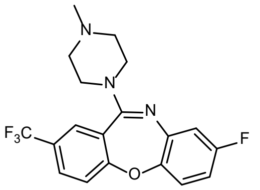

Synthesized according to GP7 from **22** (50 mg, 0.158 mmol) and 1-methylpiperazine (32 mg, 0.317 mmol). Purification of the crude product by preparative HPLC yielded the title compound as a yellow solid (25 mg, 42%). ^1H NMR (300 MHz, $CDCl_3$) δ 7.68 (dd, *J* = 8.5, 1.9 Hz, 1H), 7.58 (d, *J* = 2.1 Hz, 1H), 7.35 (d, *J* = 8.5 Hz, 1H), 7.12-7.06 (m, 3H), 6.98-6.95 (m, 1H), 4.06-3.45 (m, 4H), 3.08-2.84 (m, 4H), 2.56 (s, 3H), 2.29 (br s, 1H), 2.01 (br s, 1H); $^{13}C\{^1H\}$ NMR (75 MHz, $CDCl_3$) δ 163.2, 161.5, 159.6, 159.4, 147.7, 141.6, 128.4, 127.7, 123.7, 124.1, 122.2, 120.1, 113.4, 110.9, 54.7, 47.3, 46.0; HPLC-purity (254 nm): >99%; MALDI-HRMS: *m/z* calculated for $C_{19}H_{18}F_4N_3O[M+H]^+$: 380.1381, found: 380.1377.

2-(4-(2-Chlorodibenzo[*b*,*f*][1,4]oxazepin-11-yl)piperazin-1-yl)ethan-1-ol (**4**)

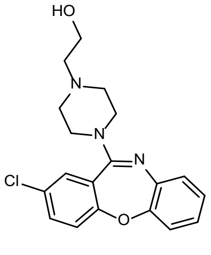

Synthesized according to GP7 from **19** (198 mg, 0.750 mmol) and hydroxyethylpiperazine (195 mg, 1.50 mmol). Purification of the crude product by preparative HPLC yielded the title compound as a brown oil and formate salt (216 mg, 72%). ^1H NMR (300 MHz, $CDCl_3$) δ 8.40 (s, 1H), 7.41 (dd, *J* = 8.6, 2.6 Hz, 1H), 7.31 (d, *J* = 2.6 Hz, 1H), 7.19 (d, *J* = 8.6 Hz, 1H), 7.16-7.07 (m, 3H), 7.05-6.99 (m, 1H), 6.51 (br s, 2H), 3.85-3.81 (m, 2H), 3.74 (br s, 4H), 2.98 (br

s, 4H), 2.89-2.85 (m, 2H); $^{13}C\{^1H\}$ NMR (75 MHz, $CDCl_3$) δ 166.8, 159.3, 158.3, 151.7, 139.6, 133.0, 130.5, 128.8, 127.1, 125.9, 125.1, 124.5, 122.9, 120.2, 59.7, 56.9, 52.2, 45.8; HPLC-purity (254 nm): 99%; MALDI-HRMS: *m/z* calculated for $C_{19}H_{21}ClN_3O_2[M+H]^+$: 358.1317, found: 358.1324.

2-(2-(4-(2-Chlorodibenzo[*b,f*][1,4]thiazepin-11-yl)piperazin-1-yl)ethoxy)ethan-1-ol (5)

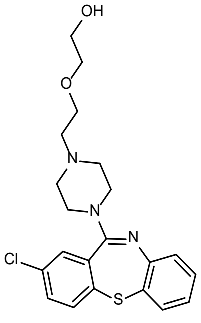

Synthesized according to GP7 from **21** (119 mg, 0.425 mmol) and 1-[2-(2-hydroxyethoxy)ethyl]piperazine (147 µL, 0.850 mmol). Purification of the crude product by preparative HPLC yielded the title compound as a brown oil and formate salt (156 mg, 79%). ^1H NMR (300 MHz, $CDCl_3$) δ 8.37 (s, 1H), 7.46-7.41 (m, 1H), 7.38 (dd, *J* = 7.7, 1.5 Hz, 1H), 7.33-7.27 (m, 2H), 7.29 (br s, 1H), 7.23-7.17 (m, 1H), 7.06 (dd, *J* = 8.0, 1.5 Hz, 1H), 6.93 (td, *J* = 7.3, 1.5 Hz, 1H), 3.88 (br s, 2H), 3.75 (t, *J* = 5.1 Hz, 2H), 3.70 (t, *J* = 4.8 Hz, 2H), 3.58 (t, *J* = 4.8 Hz, 2H), 3.55 (br s, 2H), 3.08-3.00 (m, 2H), 2.90 (t, *J* = 5.3 Hz, 2H), 2.90-2.82 (br s, 2H); $^{13}C\{^1H\}$ NMR (75 MHz, $CDCl_3$) δ 166.7, 158.9, 148.2, 138.1, 134.9, 134.8, 133.4, 132.9, 131.2, 129.4, 128.6, 127.3, 125.3, 123.6, 72.6, 66.1, 61.4, 57.2, 52.0, 45.1; HPLC-purity (254 nm): 95%; MALDI-HRMS: *m/z* calculated for $C_{21}H_{25}ClN_3O_2S[M+H]^+$: 418.1351, found: 418.1350.

2-(2-(4-(2-Chlorodibenzo[*b,f*][1,4]oxazepin-11-yl)piperazin-1-yl)ethoxy)acetic acid (6)

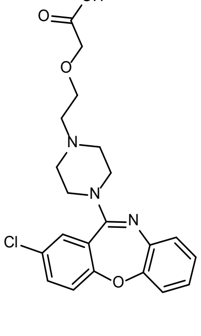

Synthesized according to GP8 from commercially purchased amoxapine (TCI, Product Number: A2499) (478 mg, 1.56 mmol), 2-(2-chloroethoxy)acetic acid (432 mg, 3.12 mmol)

and triethylamine (2.2 mL, 15.6 mmol). Purification of the crude product by flash chromatography (DCM/MeOH 95:5 to 9:1) yielded a colorless oil, which was dissolved in DCM and concentrated under reduced pressure to yield the title compound as a colorless solid (580 mg, 90%). ^{1}H NMR (300 MHz, $CDCl_3$) δ 8.24 (br s, 1H), 7.41 (dd, J = 8.7, 2.4 Hz, 1H), 7.34 (d, J = 2.4 Hz, 1H), 7.18 (d, J = 8.7 Hz, 1H), 7.15-6.98 (m, 4H), 5.91 (br s, 2H), 4.23 (br s, 2H), 4.44-3.94 (m, 6H), 3.87-3.72 (m, 6H); ^{13}C{^{1}H} NMR (75 MHz, $CDCl_3$) δ 168.8, 164.7, 159.3, 159.0, 151.6, 139.1, 133.4, 130.8, 128.7, 127.2, 126.0, 125.7, 123.9, 123.0, 120.3, 61.4, 59.5, 55.8, 41.4; HPLC-purity (254 nm): 99%; MALDI-HRMS: *m/z* calculated for $C_{21}H_{23}ClN_3O_4$ [M+H]$^+$: 416.1372, found: 416.1375.

2-(2-(4-(2-(Trifluoromethyl)dibenzo[*b,f*][1,4]oxazepin-11-yl)piperazin-1-yl)ethoxy)acetic acid (**7**)

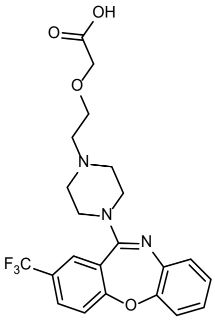

Synthesized according to GP8 from **23** (75 mg, 0.102 mmol), 2-(2-chloroethoxy)acetic acid (28 mg, 0.205 mmol) and triethylamine (0.14 mL, 1.02 mmol). Purification of the crude product by flash chromatography (DCM/MeOH 95:5 to 9:1) yielded the title compound as a colourless solid (28 mg, 61%). ^{1}H NMR (400 MHz, $CDCl_3$) δ 7.66 (dd, J = 8.5, 2.0 Hz, 1H), 7.56-7.56 (m, 1H), 7.31 (d, J = 8.5 Hz, 1H), 7.12-7.03 (m, 3H), 6.97 (td, J = 7.6, 1.8 Hz, 1H), 4.21 (s, 2H), 3.73-3.43 (m, 12H); ^{13}C{^{1}H} NMR (101 MHz, $CDCl_3$) δ 168.7, 163.4, 158.8, 151.5, 139.7, 128.5, 127.8, 127.4, 126.3, 125.4, 124.1, 123.6, 122.5, 120.5, 73.8, 69.7, 61.8, 44.3, 41.7; HPLC-purity (254 nm): 98%; MALDI-HRMS: *m/z* calculated for $C_{22}H_{23}F_3N_3O_4$ [M+H]$^+$: 450.1635, found: 450.1637.

2-(2-(4-(2-(Trifluoromethyl)dibenzo[*b,f*][1,4]oxazepin-11-yl)-1,4-diazepan-1-yl)ethoxy)acetic acid (**8**)

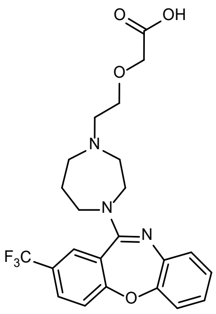

Synthesized according to GP8 from **24** (40 mg, 0.111 mmol), 2-(2-chloroethoxy)acetic acid (31 mg, 0.221 mmol) and triethylamine (0.16 mL, 1.11 mmol). Purification of the crude product by flash chromatography (DCM/MeOH 95:5 to 9:1) yielded the title compound as a colourless solid (20 mg, 39%). ^{1}H NMR (400 MHz, $CDCl_3$) δ 7.95-7.91 (m, 1H), 7.80-7.77 (m, 1H), 7.59 (t, J = 8.8 Hz, 1H), 7.22-7.18 (m, 1H), 7.11-6.93 (m, 3H), 4.63 (d, J = 4.8 Hz, 1H), 4.18-4.09 (m, 3H), 3.77-3.36 (m, 10H), 2.08-1.46 (m, 2H); ^{13}C{^{1}H} NMR (101 MHz, $CDCl_3$) δ 168.6, 162.4, 157.6, 150.7, 140.3, 129.9, 126.6, 126.3, 126.0, 126.0, 123.7, 123.6, 123.7, 122.4, 120.2, 72.5, 69.1, 68.9, 68.2, 67.6, 63.2, 60.0, 44.0; HPLC-purity (254 nm): 95%; MALDI-HRMS: *m/z* calculated for $C_{23}H_{25}F_3N_3O_4$ $[M+H]^+$: 464.1792, found: 464.1788.

References for Supporting Information

[1] D. Usoskin, A. Furlan, S. Islam, H. Abdo, P. Lonnerberg, D. Lou, J. Hjerling-Leffler, J. Haeggstrom, O. Kharchenko, P. V. Kharchenko, S. Linnarsson, P. Ernfors, *Nat. Neurosci.* **2015**, *18* (1), 145, https://doi.org/10.1038/nn.3881.

[2] N. Sharma, K. Flaherty, K. Lezgiyeva, D. E. Wagner, A. M. Klein, D. D. Ginty, *Nature* **2020**, *577* (7790), 392, https://doi.org/10.1038/s41586-019-1900-1.

[3] J. Kupari, D. Usoskin, M. Parisien, D. Lou, Y. Hu, M. Fatt, P. Lonnerberg, M. Spangberg, B. Eriksson, N. Barkas, P. V. Kharchenko, K. Lore, S. Khoury, L. Diatchenko, P. Ernfors, *Nat. Commun.* **2021**, *12* (1), 1510, https://doi.org/10.1038/s41467-021-21725-z.

[4] D. Tavares-Ferreira, S. Shiers, P. R. Ray, A. Wangzhou, V. Jeevakumar, I. Sankaranarayanan, A. M. Cervantes, J. C. Reese, A. Chamessian, B. A. Copits, P. M. Dougherty, R. W. t. Gereau, M. D. Burton, G. Dussor, T. J. Price, *Sci. Transl. Med.* **2022**, *14* (632), eabj8186, https://doi.org/10.1126/scitranslmed.abj8186.

[5] A. M. Griffin, K. M. Kahlig, R. J. Hatch, Z. A. Hughes, M. L. Chapman, B. Antonio, B. E. Marron, M. Wittmann, G. Martinez-Botella, *ACS Med Chem Lett* **2021**, *12* (4), 593, https://doi.org/10.1021/acsmedchemlett.0c00675.

[6] A. Tjaden, A. Chaikuad, E. Kowarz, R. Marschalek, S. Knapp, M. Schroder, S. Muller, *Molecules* **2022**, *27* (4), 1439, https://doi.org/10.3390/molecules27041439.

[7] A. Tjaden, R. T. Giessmann, S. Knapp, M. Schroder, S. Muller, *STAR Protoc* **2022**, *3* (4), 101791, https://doi.org/10.1016/j.xpro.2022.101791.
